# General Anæsthesia

**Published:** 1892-08

**Authors:** E. J. Buck

**Affiliations:** Warren, Mass.


### GENERAL ANÆSTHESIA.[1]

BY E. J. BUCK, WARREN, MASS.

To treat the above subject intelligently, it is necessary to present many points with which you are perfectly familiar; facts obtained both by experience and from authority. I may not give you much that is really new, except a few methods and experiences that I have found useful and interesting.

For the term as used to designate the peculiar state under consideration we are indebted to Dr. Oliver Wendell Holmes. Its literal meaning is a want of feeling or sensation. Exactly where to draw the line between symptoms induced by alcohol, ether, chloroform, opium, and its derivatives, cocaine and many other kindred drugs, their actions upon the system resembling each other perhaps only in the ultimate result, coma, may be considered merely technical. Allowing the significance of the term, I should certainly eliminate some that are now enumerated in the list.

Its history would be one of extreme interest had we facts

---

[1] Read before the Connecticut Valley Dental Society at their annual meeting held at Greenfield, Mass., January 1, 2, and 3, 1892.

instead of the vague and almost mythological references found in ancient and modern literature. So far as I am able to say, it began when Sir Humphry Davy, about the year 1800, attempted a scientific investigation of nitrous oxide and its actions upon the human system. His researches were cut short by other, and what seemed to him more important, duties. Thus, for another half-century humanity was compelled to writhe under the knife and die of maladies that are now, through the use of anæsthesia, considered amenable to surgical treatment. At that time he made the following suggestion: "As nitrous oxide in its extreme operation appears capable of destroying physical pain, it may possibly be used to advantage during surgical operations in which no great effusion of blood takes place."

In 1844, Dr. Horace Wells, of Hartford, Conn., by the merest accident, discovered, or rather observed, some of its properties; and, following up the hint, at his own suggestion submitted to a trifling operation under the influence of the gas. He was so well pleased with the result that he at once commenced to experiment upon any and all who would inhale it. In all probability he was entirely ignorant of its physiological action, or that any danger might accompany its use.

My personal experience with the agent has been as limited as I could possibly make it. One of the early theories of its action (I do not recall the author) was that of a spasmodic contraction of the pulmonary blood-vessels through the vaso-motor nerve, and was based upon the result of several post-mortem examinations, in which the right cavities of the heart were found gorged with blood, while the left auricle and ventricle were entirely empty,—a condition of the parts probably brought about by some cardiac lesion, resulting in immediate and fatal syncope.

The more recent researches of Dr. H. C. Wood lead us to very different views as to its direct actions upon these organs, while the final results remain the same. He says, "The knowledge we have of the physiological action of nitrous oxide indicates that it has no inherent anæsthetic properties, but that the loss of consciousness which follows its inhalation is the result of asphyxia, and that there is not only no proof that nitrous oxide has the power of yielding oxygen to the tissues of the body, but our present knowledge indicates that it escapes from the lungs unchanged."[1] It does not seem possible that any operator would pronounce the

---

[1] *Therapeutic Gazette.*

condition other than asphyxia, or that he would willingly inhale the gas after witnessing its action upon others.

"It has been demonstrated that animals live no longer in an atmosphere of pure nitrous oxide than they do in one of nitrogen, and that the latter will produce symptoms very nearly resembling those of nitrous oxide; that a mixture of nitrous oxide and oxygen will support life indefinitely, and finally that pure nitrous oxide is alike fatal to animal and vegetable life."[1]

Other facts brought out by the same author, and also by MM. Jolyet and Blanche, relate to pulse changes, arterial blood-pressure, and to the chemical changes which take place in the blood itself during its inhalation. Personally, I have observed that during the first stages the pulsations were reduced in frequency, while they were increased in force, and followed later by a very rapid and feeble movement, which in their experiments were accounted for by a stimulation of the inhibitory nerves. It was found that the changes in blood-pressure were enormous and rapid. First an increase in some cases to nearly twice the normal, followed by a progressive fall to zero, and were attributed to vaso-motor stimulation, followed by vaso-motor paralysis. The last symptoms are of the gravest importance in the practical exhibition of the gas, where it is known or suspected that lesions of the circulatory apparatus or blood-vessels exist, such as fatty degeneration, aneurism, or a tendency to apoplexy.

In concluding his report, Dr. Wood says, "The changes produced by nitrous oxide and nitrogen resemble somewhat those produced by mechanical asphyxia, and what difference does exist is not sufficient to convince us that nitrous oxide and nitrogen act as direct anæsthetics, and that the whole mass of evidence is concordant in showing that it is due to shutting off the supply of oxygen from the nerve-centres, and not to the accumulation of carbonic acid."[2]

The direct analysis of the blood seems to show very uniformly a displacement of oxygen in the tissues by the gas inhaled, accompanied by a marked decrease in amount of carbonic acid. As previously stated, nitrous oxide suffers no chemical change from inhalation; and since it parts with none of its oxygen, the formation of carbonic acid must necessarily stop.

There are some very grave after-effects apparently attributable to the inhalation of nitrous oxide. Just how they are induced is

---

[1] *Therapeutic Gazette.*　　[2] Ibid.

not so apparent. Several observers have found large quantities of sugar in the urine of patients after the inhalation of the gas, and from good authority come cases of permanent impairment of health dating from the same, such as menstrual suppression, epilepsy, diabetes, albumen urea, and indeed pulmonary affections, with one or more cases of miscarriage. I believe the inhalation of the gas is not without possible danger in all cases, and that it should be prohibited with pregnant women, and with young girls from twelve to seventeen years old. With persons subject to any cardiac affections it should be used with the utmost caution, if not prohibited entirely; with a patient having any renal trouble, with an habitual drunkard, or where there is an extreme nervous depression. In any case, it should only be administered by those who are perfectly familiar with its different phases, and who are able to apply prompt and intelligent measures, should occasion require. In our choice of an anæsthetic agent there is no good reason why we should select nitrous oxide in preference to sulph. ether, which we will now consider.

Ether was first discovered in the year 1540 by a German physician. Dr. Morton, of Boston, however, was the first to make a practical use of it as an anæsthetic. Unlike the discoveries of Dr. Wells, it was the result of a scientific comparison of alcohol and ether, as to what was known of the former and the possible action of the latter. After witnessing its effects upon his dog, he tried the drug upon himself, and probably was the first person to experience anything like its full effect.

Notwithstanding the low specific gravity of ether, its vapor is much more dense, and therefore heavier than common air. This is a fact we must not forget when necessary to operate by artificial light. We will pass its chemical and physical properties and consider its physiological action. Of this, unfortunately, we know but little. Alcohol has long been known to possess anæsthetic properties. Experimentally, it coagulates albumen by abstraction of water. The same is true with ether; but if this phenomenon has anything in common with the molecular changes in the nerve-centres during anæsthesia, it has not been sufficiently demonstrated to become a certainty.

The apparent order in which its action is exerted was first described by Flourens, in 1847. First, the cerebrum, resulting in loss of intelligence; second, the cerebellum, with loss of co-ordination of movement; third, the spinal cord, with loss of power to receive sensory impressions, and of initiating movements; fourth, the medulla, with paralysis of the respiratory and circulatory functions.

The treatment of symptoms and conditions, about which so little reliable information is at hand, or possible to obtain, is not a little difficult, and renders the subject open to the widest theories and speculation. It would seem proper, in the first instance, to refer to the phenomena of natural sleep. The refreshing influence and imperial necessity of such repose points clearly to the restorative processes going on within the body. Such a state is properly viewed as a normal condition, and yet how very nearly do some of its symptoms and outward appearances resemble what we term pathological coma. It has been demonstrated, in many ways, that during natural sleep the volume of blood in the brain is much diminished. The action of the heart and lungs is retarded, with a marked lowering of the temperature. Observe an infant. On going to sleep the scalp at the fontanels will be found to protrude for a short time, but when the deepest slumber is reached, just the opposite condition prevails. If we disturb it in the slightest degree, more or less evidence of an increased cerebral circulation is apparent. It seems probable that the above conditions are a natural sequence of sleep, rather than that sleep is a result of the condition. Just here the analogy must stop. There are, however, many highly interesting phenomena associated with natural sleep that are not pertinent in this place, but well worth reading.

We are compelled to draw other conclusions from the study of different forms of pathological sleep. In pronounced cerebral hyperæmia the cortical portions, together with the membranes and immediate vicinity of the large arteries, are found to be variously distended. As a natural consequence, the veins will also be found to be more or less strangulated and containing large quantities of venous blood. The above morbid condition would result, first, in depriving the deeper portions of an amount of blood sufficient for their needs; and second, those portions in the immediate vicinity of the greatest congestion would soon suffer from the vitiated condition of the blood supplied.

Anæmia of the deeper tissues must, and in fact does, accompany any considerable congestion of the cortex. How this is to be explained other than by strangulation of the deeper vessels is not easy to say. Hammond enumerates, among other symptoms of cerebral hyperæmia, confusion of ideas, hallucinations, morbid apprehensions of impending evil, furious delirium, profane and obscene language. If these are not sufficiently suggestive, we may add tinnitus aurium, the peculiar flushing of the face, and occasionally perspiration. The last symptom never occurs in the earlier stages of ether anæsthesia, and would seem to be nature's attempt to restore a normal condition. A study of the graver forms of cerebral irritation reveals the same train of symptoms. To those who have observed the symptoms of apoplexy, where considerable extravasation exists, the analogy becomes very marked.

While the outward appearances and actual conditions in ether anæsthesia so closely resemble cerebral irritation, we must recognize some subtile, primordial influence upon the nerve-centres, by which these conditions are brought about. What that is, or how it is exerted, seems to me of little account, since we do not look in that direction for a failure of the vital forces, and the length of time during which a person in perfect health would endure the toxic effect of ether would be measured by the needs of the nervous tissues for actual nourishment. Experience teaches that with ether, as with nitrous oxide, the greatest danger is from paralysis of the respiratory nerves.

It is very difficult to define the successive stages of ether anæsthesia; some authors describing two, some three, others four. Of course its action is progressive, the patient passing through a first stage, or one of exhilaration, before losing consciousness or the power to reason. I would term the latter a second, or stage of dreams, for it is now that he receives those impressions which are often the subject of confused comment immediately after waking. What has been described as a third or spasm stage I would include with the second, the symptoms being the same except in degree, and seem to be the very last evidence of cerebral function.

Finally we reach the third, or paralytic stage, the very first part of which is true surgical anæsthesia, and should be reached before an operation of any magnitude is attempted. There is a time, however, during the very last part of the first stage, when minor operations requiring but little time may be accomplished without pain, and in many cases without the knowledge of the patient. (The safety of this practice, however, has been questioned.)

What we may say or think concerning the preparation of the patient amounts to but little, since that must depend wholly upon the character and urgency of the case; but for obvious reasons ether should not be inhaled immediately after a full meal. It is much better to wait from six to eight hours, and about half an hour before the operation a glass of milk or coffee may be taken; not for the nourishment to be derived from it, but to dilute the constant stream of ether-laden saliva which will be swallowed during the first stages of anæsthesia, and which, acting as a local irritant upon the gastric nerves, will produce unpleasant results in most cases. If we can avoid this complication with the danger accompanying it, of particles of solid food being drawn into the air-passages, we have gotten rid of one of its greatest dangers.

I see no reason for giving alcoholic stimulants except in cases of extreme nervous depression. In case of shock, it might, as a special remedy directed to that condition, be administered. In such cases, unless an immediate operation were deemed necessary, I think you would prefer to wait for a reaction. The patient's clothing must not interfere with the circulation or easy respiration, and it should be so adjusted that it can be easily removed if necessary to reach the surface of the body. When possible, the temperature of the room should be raised to 70° or 80°, as it has an important bearing upon the rapidity of evaporation, and hence completion of anæsthesia. It will also assist in maintaining the temperature of the patient, which, owing to "retardation of tissue-changes and the absence of the normal amount of oxygen admitted to the circulation, is considerably lowered, and may suddenly become dangerously low." If, after we have studied our patient's condition, we deem it safe to administer the drug, his position must next occupy our attention. In this, too, we must be governed by the nature of the operation, never forgetting the possible dangers arising from cardiac and respiratory failure, together with danger of asphyxia from mechanical causes. Whatever position is chosen, we may meet with many difficulties that will require skill and judgment to surmount. Any unnecessary noise—such as talking, whispering, or laughing in the room—should be stopped at once, and no one but the operator speak to the patient. If it becomes necessary to use force to limit his movements, let it be applied promptly, otherwise it is much better to let him take his own course.

Of the many immediate dangers attending ether anæsthesia, statistics show that asphyxia, either mechanical or from failure of respiration, is the greatest. During the first and second stages the mucous surface of the mouth and pharynx, together with the salivary glands, secrete large quantities of their respective fluids, which may find their way into the larynx, giving the patient much discomfort and often producing dangerous symptoms. "If the accumulation becomes too great, it is much safer to suspend anæsthesia until the throat is thoroughly cleared, or the patient is relieved by his own efforts." If by chance any foreign body should enter the larynx, and the patient be unable to dislodge it by the violent coughing it produces, we have a very unfortunate condition, the sequel of which will depend upon the operator's skill and courage.

In the third stage the tongue, becoming paralyzed, may fall back upon the epiglottis, thus interfering with the act of respiration. For the relief of this condition various methods may be practised, but that suggested by Dr. Howard will give the best results. The main features of this method are as follows:

"Place the patient upon his back" (preferably upon the floor), "elevating the thorax by placing a roll of blankets beneath the scapulæ. In this position the chin will present to the ceiling instead of the face, so changing the line of gravitation that the tongue will fall from the pharynx and rest upon the hard palate. The entire posterior wall of the pharynx will be shifted backward, at the same time the larynx will be pulled downward and forward by the sterno-hyoid muscles; while the lower jaw, being lifted upward and forward, will cause a tension of the mylo- and genio-hyoid muscles, thus lifting the hyoid bone, and through the hyo-epiglottic ligament cause the epiglottis to share the same movement."[1] A few inhalations of air will restore the functions of the parts.

If we have escaped the preceding complications, our patient may suddenly and without warning stop breathing entirely from paralysis of the respiratory centres. The face becomes livid with rapidly increasing signs of asphyxia, the muscular system is completely relaxed, the pulse being reduced in frequency and force. The absence of muscular rigidity will differentiate this condition from one of simple spasm, in which respiration is voluntarily resumed in a few seconds.

No time should be lost in meeting this difficulty, either with the battery or artificial respiration, by one of the different methods. The position of Howard will favor whichever method is employed.

"One of the characteristic effects of ether is that in its progressive action upon the vital functions the respiratory centres become depressed more rapidly than the circulatory, the heart continuing to beat for some time after respiration has ceased." The greatest danger, as before stated, is to be found in this direction. Both the pulse and respiration should be closely watched, and any failure of the former will be found to have been preceded by an unmistakable depression of the latter. While cardiac syncope is very liable to occur in chloroform narcosis, happily it is very rare with ether.

---

[1] "American System of Dentistry."

Should we be so unfortunate as to be overtaken with an accident of this kind, I know of no reliable symptom that will warn us of its approach. It is certain that well-defined indications of cerebral anæmia will announce its presence, if not discovered sooner, and to this symptom our efforts should be directed by placing the patient in such a condition that by gravitation a fresh supply of blood will reach the brain. We should make all possible endeavor to re-establish respiration, and, by well-directed manipulation over the cervical veins, assist the return flow of venous blood. Experiments with amyl nitrate have demonstrated its value as a remedy in cases of cerebral anæmia. This is supposed to be derived from its power to control many spasmodic affections, and by relaxing the muscular coats of the cerebral vessels it assists in restoring circulation. Given in doses of two to four minims, it is said to raise the temperature of the body.

There are, perhaps, but few conditions in which ether anæsthesia would be entirely inadmissable. Those would include some of the more serious cardiac troubles, especially if the patient is very old. When the action of the lungs is considerably limited, or where there is an excessive secretion from irritated surfaces along the respiratory tract, we must be very cautious. Drunkards, opium-eaters, or persons who are known to faint from slight causes, as well as cases of advanced pregnancy, must be treated always as dangerous.

The after-effects of ether anæsthesia, so far as it is known, are very few. More or less pulmonary congestion must always follow, causing with some patients a slight cough, which may last from one to two days. The patient must be cautioned against undue exposure, otherwise, if the conditions be right, pneumonia may result. The severe headache produced by cerebral congestion may be relieved by appropriate treatment, and, unless the patient be advanced in years or has a tendency to apoplexy, is not a grave symptom. The ever-present nausea, a sympathetic manifestation of the preceding condition, is one of the most distressing and objectionable features of ether narcosis. If the patient remains supine and is allowed to sleep off the drug, it will generally disappear as the normal circulation is resumed. Chloroform given internally in doses of two to four minims will relieve and often control the most persistent vomiting. I have used, also, pure creosote in one-minim doses, both of which act as a local anæsthetic upon the gastric nerves, thus tiding over the difficulty until other conditions prevail.

Finally, the patient should be closely watched until he has completely recovered, for even now the vital forces may suddenly fail.

Much can be said of chloroform, but after we had finished we should be compelled to add, don't use it. The sphygmographic tracing of a patient under its influence says, in unmistakable terms, do not use it. It is said to have been discovered by a Dr. Guthrie, formerly of Brimfield, Massachusetts, in 1831, and independently in 1832 by Liebig. It seems to unite with both alcohol and ether, but when such a combination is evaporated from a sponge we have no evidence that they remain combined in the form of vapor. On the contrary, the vapor is at first strongly impregnated with the odor of ether, and later on we lose nearly all trace of it. We should expect this result, since its specific gravity is nearly twice that of ether. If we derive any benefit by such a combination, it must be from the fact that alcohol retards the evaporation of the chloroform and insures a greater admixture of air. There must come a time, however, when the patient, already partially anæsthetized with ether, is inhaling nearly pure chloroform, and at a time when he is in no condition to endure its depressing influence. When taken alone, or in combination, its action is more rapid and less under our control than ether. Its powerful action upon the vaso-motor nerve-centres reduces the arterial blood-pressure to an alarming extent, and just here we may look, perhaps, for some clue by which we may trace back to a cause some of the sudden and mysterious deaths which investigation and post-mortems have given us no hint of the truth. If we will remember the relative position of the pneumogastric and vaso-motor centres, and also their many reflex connections with other centres, we can readily see why any injury to one is at once reflected to others.[1] "The blood contained in the veins is useless for the nutrition of the tissues. It is supplied and retained in the arteries, first, by fresh blood being pumped into them from the venous system; and secondly, by the contraction of the capillaries, which prevents it from passing too rapidly again to the veins. Any shock or injury, such as extraction of a tooth, will be reflected to both centres; and if the heart's action is thereby diminished or stopped temporarily, the same irritation will cause a contraction of the arterioles, thus retaining the blood and furnishing nourishment to the tissues until the action of the heart is resumed. In complete anæsthesia the reflex action of both nerves is abolished; but in partial anæsthesia the vaso-motor centre may become insensible

[1] "American System of Dentistry."

before the vagus, and any sufficient injury might stop the supply, while that in the arteries flows on to the venous side," where we find it always in case of shock and in the cadaver.

I wish to add, in relation to ether narcosis, I have observed that if inhaled in the early morning, while the stomach and its dependent nerves are in a state of perfect rest, we shall have less nausea; also if we use Squibb's ether and conduct our manipulations so as to reach perfect anæsthesia in the shortest possible time, the same good results will obtain. Just here you may say is a difficulty,—that of strangling our patient. But let us impress upon his mind and teach him the importance of breathing fully and deeply through his nose before we commence. One who has not tried it has no idea how kindly the nares and upper pharanx will take to its fumes, and of the agreeable sensation, when compared to that of inhaling by the mouth.

I have already taken too much of your time, but I have met with several cases in my practice, in which I took a very vivid interest, and after what I have written it might not be improper to give a brief account of the same.

Case I.—Male; age, thirty-five to forty. Temperament, nervous; habits, intemperate. Was treated for neuralgia in a palliative way, with morphia and chloral, for four months. At last his physician sent him to me to discover a cause, which was located in a closed, chronic alveolar abscess. After inhaling nitrous oxide a very short time, he suddenly showed marked symptoms of asphyxia; voluntary respiration ceased almost immediately, with pulse barely perceptible. Since the object was to remove the tooth, I did so, hoping to derive a benefit from the shock and accomplish the object also. (After deliberate thought, however, I cannot condemn my course in this respect too severely.) Applied Howard's method and succeeded, after a time, in restoring normal respiration.

Case II.—Male; age, twenty-five. Has no peculiarities. Temperament, phlegmonous. Ether was the anæsthetic, and result asphyxia, by the tongue falling upon the epiglottis. In this case much time was lost in trying to draw the tongue forward, which failed, owing to the titanic contraction of the facial muscles, until all voluntary respiration had ceased. I at last placed him upon his back, raising the thorax about six inches, allowing the head to rest upon the floor. In this case I used direct insufflation.

Case III.—Female; age, thirty-five. Temperament bilious, rather anæmic; had recently suffered both mentally and physically until the nervous system was somewhat reduced. Ether was administered very cautiously. A condition of asphyxia followed, with entire loss of respiration. Heart's action very weak. Treatment same as Case II.

In studying these cases we discover two distinct conditions: Cases I. and III., respiratory failure; Case II., asphyxia from mechanical cause. I cannot say that the last medicine cured my patients or saved their lives, but I certainly know that their condition was very serious.

